# Lysine and arginine methylation of transcription factors

**DOI:** 10.1007/s00018-024-05531-6

**Published:** 2024-12-16

**Authors:** Benedetto Daniele Giaimo, Francesca Ferrante, Tilman Borggrefe

**Affiliations:** Institute of Biochemistry, Justus-Liebig-University Giessen, Friedrichstrasse 24, 35392 Giessen, Germany

**Keywords:** KMT, KDM, PRMT, PTM, Hypoxia, Cell cycle

## Abstract

Post-translational modifications (PTMs) are implicated in many biological processes including receptor activation, signal transduction, transcriptional regulation and protein turnover. Lysine’s side chain is particularly notable, as it can undergo methylation, acetylation, SUMOylation and ubiquitination. Methylation affects not only lysine but also arginine residues, both of which are implicated in epigenetic regulation. Beyond histone-tails as substrates, dynamic methylation of transcription factors has been described. The focus of this review is on these non-histone substrates providing a detailed discussion of what is currently known about methylation of hypoxia-inducible factor (HIF), P53, nuclear receptors (NRs) and RELA. The role of methylation in regulating protein stability and function by acting as docking sites for methyl-reader proteins and via their crosstalk with other PTMs is explored.

## Introduction

Post-translational modifications (PTMs) of proteins on specific residues provide a sophisticated mechanism for regulating their activity, function and/or localization. Several residues are subjected to be modified, for example phosphorylation occurs on serine (S), threonine (T) and tyrosine (Y) residues. Lysine (K) residues are for example modified by acetylation, methylation, SUMOylation and ubiquitination, while arginine (R) residues are modified by methylation. A lot of research has been devoted to characterize the regulation of PTMs that regulate histones, which constitute the building blocks of the chromatin however, much less is known about PTMs of non-histone proteins. When considering methylation of non-histone proteins such as transcription factors our knowledge is even more scarce. This review explores the role of arginine and lysine methylation in transcription factors, highlighting how these PTMs influence their activity. Additionally, we also discuss arginine and lysine methylation in the context of regulating DNA methyltransferases (DNMTs) activity.

Protein methylation can occur on lysine or arginine residues as a result of the activity of lysine methyltransferases (KMTs) and protein arginine methyltransferases (PRMTs), respectively that use the *S*-adenosyl-l-methionine (SAM) as donor of the methyl group.

**Fig. 1:**
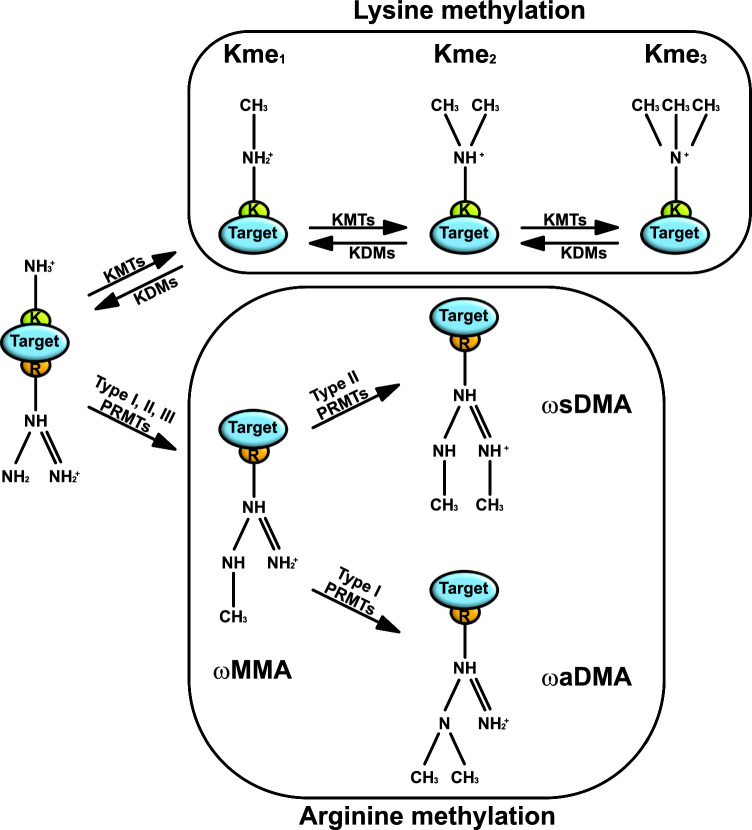
Schematic representation of lysine (K) and arginine (R) methylation. The NH_3_^+^ group of lysine (K) residues can be methylated by lysine methyltrasferases (KMTs) or demethylated by lysine demethylases (KDMs). Accordingly, to the degree of methylation, it is possible to distinguish three different methylation states: monomethylation (Kme_1_) with only one CH_3_ group; dimethylation (Kme_2_) with two CH_3_ groups; trimethylation (Kme_3_) with three CH_3_ groups. Arginine (R) residues are methylated by meaning of mono- and dimethylation by protein arginine (R) methyltransferases (PRMTs). Arginine monomethylation (indicated as ωMMA) is catalyzed by Type I (PRMT1, 2, 3, 4, 6 and 8), Type II (PRMT5 and 9) and Type III (PRMT7) PRMTs. Arginine dimethylation can occur as symmetrical (indicated as ωsDMA) or asymmetrical (indicated as ωaDMA): Type I catalyze ωaDMA whereas Type II (PRMT5 and 9) catalyze ωsDMA. Type III PRMT catalyze exclusively ωMMA

KMTs transfer the methyl group from the SAM to the ε-amino group of the lysine of the target protein which can accept up to three methyl groups generating mono-, di- or trimethylation of lysine residues (Kme_1_, Kme_2_ and Kme_3_, respectively; Fig. 1). KMTs can be classified in two different classes (Table 1): One class contains a highly conserved SET [Su(var)3–9, Enhancer of Zeste, and Trithorax] domain and are usually involved in the methylation of lysine residues located in the N-terminal tails of the histones; The members of the other class do not have a SET domain and are involved in the methylation of lysine residues located within the histone globular core, this class is characterized by the highly conserved KMT4 family [1, 2].

**Table 1:** List of lysine methyltransferases (KMTs) and protein arginine methyltransferases (PRMTs)

	Histone target (s)	Non-histone target (s)	Inhibitor(s)	References
*KMTs*				
EEF1AKMT4	ND	ND	NP	
KMT1A/SUV39H1	H3K9	ND	F5446	[134]
KMT1B/SUV39H2	H2AXK134, H3K9	ND	OTS186935, OTS193320	[135]
KMT1C/EHMT2/G9A	H1.2K187, H1.4K26, H3K9, H3K18, H3K23, H3K27, H3K56	C/EBPβ, DNMT3A, RUNX3, MEF2D, MYOD1, P53	BIX-01294, UNC0224, UNC0638	[43, 97, 107, 115, 136–140]
KMT1D/EHMT1/GLP	H3K9, H3K18, H3K23, H3K27	DNMT3A, P53	BIX-01294, UNC0224, UNC0638	[43, 115, 136–138, 141]
KMT1E/SETDB1	H3K9	ND	SETDB1-TTD-IN-1	[142]
KMT1F/SETDB2	H3K9	ND	NP	
KMT2A/MLL1	H3K4	ND	MM-401, Win6mer	[143, 144]
KMT2B/MLL2/MLL4/ WBP7	H3K4	ND	NP	
KMT2C/MLL3	H3K4	ND	NP	
KMT2D/MLL2/MLL4	H3K4	ND	C1, C16	[145]
KMT2E/MLL5/SETD5B	H3K4	ND	NP	
KMT2F/SETD1A	H3K4, H3K37	ND	Win6mer	[144]
KMT2G/SETD1B	H3K4	ND	NP	
KMT2H/ASH1L	H3K4, H3K36	ND	AS-99	[146]
KMT3A/SETD2	H3K36, H3K37	ND	EPZ-719, EZM0414	[147, 148]
KMT3B/NSD1	H3K36, H4K20	RELA	BT5	[73, 78, 149]
KMT3C/SMYD2	H3K4, H3K36	AR, ERα, P53	AZ505, BAY-598, LLY-507	[25, 57, 58, 69, 150–152]
KMT3D/SMYD1	H3K4	ND	NP	
KMT3E/SMYD3	H3K4, H4K5	ND	BCI-121, EM127, EPZ031686	[153–155]
KMT3F/NSD3	H3K36	ND	13i, BI-9321	[156, 157]
KMT3G/NSD2	H3K36	ND	MR837	[158]
KMT4/DOT1L	H3K79	ND	EPZ004777, Pinometostat, SGC0946	[159–161]
KMT5A/SETD8	H4K20	P53	SPS8I1 − 3, UNC0379	[26, 39, 40, 162, 163]
KMT5B/SUV420H1	H4K20	ND	A-196	[164]
KMT5C/SUV420H2	H4K20	ND	A-196	[164]
KMT6A/EZH2	H3K27, H2BK120	GATA4, RORα, STAT3	DZNep, GSK2816126, Tazemetostat	[63, 88, 89, 119, 165–167]
KMT6B/EZH1	H3K27	ND	UNC1999, Valemetostat	[168, 169]
KMT7/SETD7	H3K4	AR, DNMT1, E2F1, ERα, FOXO3, FXR, GLI3, HIF1α, HIF2α, P53, PDX1, RELA, SOX2, STAT3, YY1, YY2	(R)-PFI-2, Set7_1a	[17–19, 22, 26, 27, 32, 48–50, 54, 55, 67, 68, 72–75, 85, 93, 95, 103, 104, 109, 112, 114, 170–175]
KMT8A/PRDM2	H3K9	ND	NP	
KMT8B/PRDM9	H3K4	ND	MRK-740	[176]
KMT8C/PRDM6	H4K20	ND	NP	
KMT8D/PRDM8	H3K9	ND	NP	
KMT8E/MECOM	H3K9	ND	NP	
KMT8F/PRDM16	H3K9	ND	NP	
SETD5	H3K36	ND	NP	
SETD6	H2A.ZK7	E2F1, RELA	NP	[51, 76, 77]
SETMAR	H3K4, H3K36	ND	NP	
*PRMTs*				
PRMT1	H2AR11, H4R3	AML1/ETO, C/EBPα, E2F1, ERα, FOXO1, GLI1, KLF4, MBD2, MYOD1, NRF2, PR, RELA, RUNX1, STAT3, TWIST1	AMI-1, GSK3368715, MS023	[53, 60, 66, 80, 92, 98–101, 110, 116, 177–185]
PRMT2	H3R8, H4R3	STAT3	NP	[91]
PRMT3	H4R3	HIF1α	GSK3368715, MS023, SGC707	[20, 177, 178, 186]
PRMT4	H3R2, H3R17, H3R26, H3R42, H4R3	PAX7, RELA, SOX2, SOX9	CARM1-IN-4, GSK3368715, MS023, MS049, PRMT4-IN-1	[83, 105, 177, 178, 187–191]
PRMT5	H2AR3, H3R2, H3R8, H4R3	AR, BCL6, E2F1, GATA4, GLI1, HOXA9, MBD2, P53, PAX3, RELA, RORα, SREBP1A	GSK3235025, GSK3326595, Onametostat	[45, 52, 53, 64, 70, 79, 81, 82, 111, 116, 192–199]
PRMT6	H2AR3, H2AR11, H2AR29, H3R2, H3R42, H4R3	FOXO3, STAT3	EPZ020411, GSK3368715, MS023, MS049	[90, 177, 178, 187, 200]
PRMT7	H2AR3, H3R2, H4R3, H4R17, H4R19	ND	SGC3027	[201]
PRMT8	ND	ND	GSK3368715, MS023	[177, 178]
PRMT9	ND	ND	LD2	[202]

Indicated are the histone and the DNA binding proteins known to be methylated by each enzyme including known inhibitors. For simplicity, we do not report all the inhibitors available on the market

*ND* Not described, *NP* Not published

PRMTs catalyze the generation of ω-N^G^-methylarginine (mono-methylarginine, ωMMA) or ω-N^G^,N^G^-methylarginine (di-methylarginines, ωDMA; Fig. 1). Importantly, DMA can be either symmetric (ωsDMA) or asymmetric (ωaDMA; Fig. 1). Based on their catalyzed reaction, mammalian PRMTs can be classified in type I, II and III class (Table 1): Type I (PRMT1, 2, 3, 4, 6 and 8) catalyze ωMMA and ωaDMA; Type II (PRMT5 and 9) catalyze ωMMA and ωsDMA; Type III (PRMT7) catalyze only ωMMA. Most of the PRMTs methylate glycine- and arginine-rich (GAR) motifs [3] however, PRMT4 methylates within proline-, glycine- and methionine-rich (PGM) motifs [4] and PRMT5 catalyzes ωsDMA within both GAR and PGM motifs [5].

For a long time, protein methylation was regarded as a stable modification until the identification of the first lysine demethylase (KDM) KDM1A/LSD1 (lysine demethylase 1A/lysine-specific histone demethylase (1) [6]. KDMs are classified in two groups (Table 2): The LSD family of flavin adenine dinucleotide (FAD)-dependent amine oxidases that includes KDM1A/LSD1 and KDM1B/LSD2 (lysine demethylase 1B/lysine-specific histone demethylase (2); and the jumonji C (JMJC) domain-containing family of KDMs that includes oxygenases that use molecular oxygen as a substrate to promote protein demethylation. While LSD family members catalyze demethylation of mono- and dimethylated lysine residues, JMJC family members can demethylate both mono-, di- and trimethylated lysine residues. The existence of arginine demethylases (RDMs) has been under debate for long time however, more evidences suggest the existence of this enzymatic function. Initial studies have observed Jumonji domain-containing 6 (JMJD6)-mediated arginine demethylation of histone and non-histone proteins both in cell-free and in vitro assays [7–11]. However, two additional studies challenged the conclusion that JMJD6 acts as an arginine demethylase as the authors could not detect such activity [12, 13]. Subsequently, Walport and colleagues observed arginine demethylase activity for KDM3A/JMJD1A (lysine demethylase 3A/Jumonji domain-containing 1A), KDM4E/JMJD2E (lysine demethylase 4E/Jumonji domain-containing 2E), KDM5C/JARID1C [lysine demethylase 5C/Jumonji AT rich interactive (ARID) domain 1C] and KDM6B/JMJD3 (lysine demethylase 4E/Jumonji domain-containing 3) in cell-free assays [13]. Finally, KDM3B/JMJD1B (lysine demethylase 3B/Jumonji domain-containing 1B) was shown to have arginine demethylase activity versus histone proteins both in cell-free and in vitro assays [14] and KDM5C/JARID1C to promote arginine demethylation of non-histone proteins in in vitro assays [15]. However more efforts are required to validate the arginine demethylase activity of these enzymes in vivo.

**Table 2:** List of lysine demethylases (KDMs)

	Histone target (s)	Non-histone target (s)	Inhibitor (s)	References
*KDMs/RDMs*				
KDM1A/LSD1	H3K4, H3K9	E2F1, HIF1α, MEF2D, STAT3, OCT4, P53, SOX2, YY2	7c, GSK-LSD1, Iadademstat, Pargyline	[18, 19, 37, 48, 49, 85, 95, 102, 104, 107, 203–205, 128]
KDM1B/LSD2	H3K4, H3K9	ND	7c	[204]
KDM2A	H3K4, H3K36	RELA	Daminozide, KDM2A/7A-IN-1	[73, 78, 206, 207]
KDM2B	H3K4, H3K36	ND	NP	
KDM3A/JMJD1A	H3K9	P53	NP	[23, 24]
KDM3B/JMJD1B	H3K9	ND	P3FI-63	[208]
KDM3C/JMJD1C	H3K9	STAT3	U193D7	[86, 87]
KDM4A/JMJD2A	H3K9, H3K36	ND	5-carboxy-8HQ, IOX1, NCGC00244536, NSC636819	[209–212]
KDM4B/JMJD2B	H3K9, H3K36	ND	NCGC00244536, NSC636819	[211, 212]
KDM4C/JMJD2C	H3K9, H3K36	ND	KDM4C-IN-1, NCGC00244536	[212, 213]
KDM4D/JMJD2D	H3K9, H3K36	ND	KDM4D-IN-1, NCGC00244536	[212, 214]
KDM4E/JMJD2E	ND	ND	3-substituted pyridine 2,4-dicarboxylate	[215]
KDM4F/JMJD2F	ND	ND	NP	
KDM5A/JARID1A	H3K4	ND	KDOAM-25, JQKD82, PBIT, Ryuvidine	[216–219]
KDM5B/JARID1B	H3K4	ND	AS8351, KDOAM-25, PBIT, Ryuvidine	[216, 217, 219, 220]
KDM5C/JARID1C	H3K4	ND	KDOAM-25, PBIT, Ryuvidine	[216, 217, 219]
KDM5D/JARID1D	H3K4	ND	KDOAM-25,	[219]
KDM6A/UTX	H3K27	ND	GSK-J1, GSK-J4	[221]
KDM6B/JMJD3	H3K27	ND	GSK-J1, GSK-J4	[221]
KDM6C/UTY	H3K27	ND	NP	
KDM7A/JHDM1D	H3K9, H3K27, H4K20	ND	Daminozide, KDM2A/7A-IN-1	[206, 207]
KDM7B/PHF8	H3K9, H3K27, H4K20	YY1	Daminozide, iPHF8	[94, 206]
KDM7C/PHF2	H3K9	ND	NP	
KDM8/JMJD5	H3K36	ND	NP	
KDM9	H4K20	ND	NP	
NO66	H3K4, H3K36	ND	NP	

Indicated are the histone and the DNA binding proteins known to be demethylated by each enzyme including known inhibitors. For simplicity, we do not report all the inhibitors available on the market

*ND* Not described, *NP* Not published

Post-translationally modified residues represent docking sites for the interaction with proteins that contain domains able to “read” modified residue(s). More domains have been described to recognize methylated lysine and/or arginine: Ankyrin (ANK) repeats, WD40 repeat domain, plant homeodomain (PHD) finger, Tudor domain, proline-tryptophan-tryptophan-proline (PWWP) domain, bromo adjacent homology (BAH) domain, malignant brain tumor (MBT) domain, ATRX-DNMT3A-DNMT3L (ADD) domain, chromodomain (CD) and zinc finger cysteine-tryptophan (zn-CW) domain.

## Methylation of transcription factors

### Hypoxia-inducible factors (HIFs)

Hypoxia-inducible factors (HIFs) are the key mediators of the hypoxia response, they function as heterodimers composed of an oxygen (O_2_)-sensitive α and an O_2_-insensitive β subunit. Under plentiful O_2_ concentrations, defined as normoxia, HIFα proteins are hydroxylated by factor inhibiting HIF [FIH; encoded by the *HIF1AN* (hypoxia-inducible factor 1-alpha inhibitor) gene] and by prolyl hydroxylase domain (PHD) proteins and their transcriptional activity is prevented. FIH-mediated hydroxylation of HIFα proteins on asparagine (*N*) residues prevents their interaction with the transcriptional activator KAT3B/EP300 (lysine acetyltransferase 3B/E1A-binding protein 300 kD) while the PHDs-mediated hydroxylation on proline (P) residues leads to their interaction with the von Hippel–Lindau tumor suppressor protein (pVHL) that finally results in the ubiquitin-dependent proteasomal degradation of HIFα proteins. Given that O_2_ is the key cofactor for both FIH and PHDs their activity is suppressed under hypoxic conditions leading to stabilization of HIFα proteins and activation of their target genes upon their heterodimerization with the HIFβ subunit(s) [16].

In the recent years, it has become increasingly clear that the regulation of HIF proteins is not exclusively dependent on their hydroxylation and ubiquitination but also on other PTMs, such as lysine methylation. KMT7/SETD7 (lysine methyltransferase 7/SET domain-containing 7) monomethylates HIF1α and HIF2α at highly conserved lysine residues (HIF1αK32me_1_ and HIF2αK29me_1_, respectively) significantly reducing their transcriptional activity [17]. KMT7/SETD7-dependent methylation of HIF1α reduces its DNA-binding activity without influencing its nuclear translocation, mRNA and protein levels [17]. Notably, the expression of KMT7/SETD7 is reduced under hypoxic conditions leading to a decrease of methylation of HIF1α at K32 [17]. Interestingly, another study found that under hypoxia, the same KMT7/SETD7-mediated methylation on K32 of HIF1α reduces its nuclear stability by promoting increased ubiquitination. This regulation is counteracted by the demethylase activity of KDM1A/LSD1. In fact, overexpression of KDM1A/LSD1 enhances the stabilization of HIF1α and reduces its ubiquitination [18]. However, a second KMT7/SETD7-mediated methylation site has been identified within HIF1α [19]: This dimethylation on K391, that is again counteracted by KDM1A/LSD1, leads to increased pVHL-mediated ubiquitination of HIF1α. In fact, KDM1A/LSD1 reduces HIF1α methylation, which in turn reduces the PHD2-mediated hydroxylation and the pVHL-mediated ubiquitination of HIF1α [19].

The regulation of the hypoxia response encompasses also the asymmetric arginine dimethylation of HIF1α on position 282 (HIF1αR282me_2a_) which is dependent on PRMT3 [20]. Interestingly, under hypoxia, PRMT3 stabilizes the HIF1α protein preventing its ubiquitination and this methylation is essential for PRMT3-mediated tumorigenesis [20]. Given that hypoxia has been shown to influence the activity of KDM5C/JARID1C finally impacting om the arginine methylation state of ATG1/UNC-51 like autophagy activating kinase 1 (ULK1) [15] it will be important to evaluate whether KDM5C/JARID1C also demethylates arginines of the HIF-proteins.

**Fig. 2:**
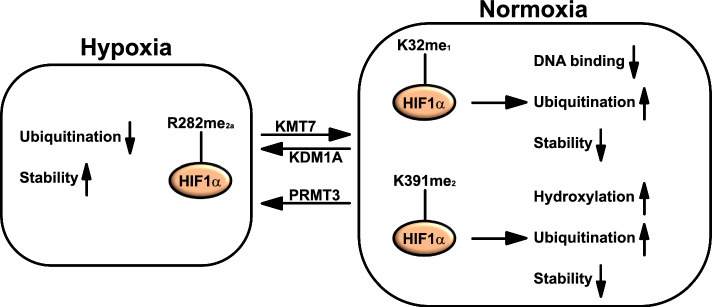
Regulation of hypoxia-inducible factor 1-alpha (HIF1α) by lysine (K) and arginine (R) methylation. Under plentiful O_2_ concentrations (defined as normoxia), KMT7/SETD7 (lysine methyltransferase 7/SET domain-containing 7) catalyzes hypoxia-inducible factor 1-alpha (HIF1α) monomethylation on K32 (HIF1αK32me_1_) and dimethylation on K391 (HIF1αK391me_2_) resulting in reduced stability of HIF1α as consequence of increased ubiquitination and degradation. K32me_1_ also leads to reduced DNA binding of HIF1α while K391me_2_ is associated with increased hydroxylation of HIF1α. Both HIF1αK32me_1_ and HIF1αK391me_2_ are demethylated by KDM1A/LSD1 (lysine demethylase 1A/lysine-specific histone demethylase 1) leading to increased stability of HIF1α. Under hypoxia, protein arginine (R) methyltransferase 3 (PRMT3) catalyzes asymmetrical dimethylation on R282 (HIF1αR282me_2a_) leading to reduced ubiquitination and as consequence increased stability of the protein. *KDM1A = KDM1A/LSD1* lysine demethylase 1A/lysine-specific histone demethylase 1; *KMT7 = KMT7/SETD7* lysine methyltransferase 7/SET domain-containing 7; *PRMT3* protein arginine methyltransferase 3

In summary, these data suggest that under normoxia, KMT7/SETD7-mediated methylation of HIF1α results in increased ubiquitination and degradation (Fig. 2). Under hypoxic conditions, demethylation of HIF1α by KDM1A/LSD1 and PRMT3-mediated methylation of HIF1α lead to its stabilization (Fig. 2).

### P53

P53 is a transcription factor which function is to protect cells from genotoxic stresses. P53 is physiologically ubiquitinated by the E3 ubiquitin ligase mouse double minute 2 (MDM2) causing its proteasomal degradation. However, upon DNA damages, P53 is phosphorylated and this prevents its interaction with MDM2 leading to P53 stabilization. As consequence, the stabilization of P53 leads to its tetramerization and this tetramer works as a transcriptional activator of several target genes which function is to promote the reparation of the DNA damages or, alternatively, induce the apoptotic response [21].

P53 is heavily regulated by a wide range of different PTMs and among them lysine methylation plays a significant role. KMT7/SETD7-mediated monomethylation on K372 (P53K372me_1_) positively regulates the function of P53 [22] while its demethylation is mediated by KDM3A/JMJD1A and leads to reduced expression of pro-apoptotic genes [23, 24]. On the other hand, KMT3C/SMYD2 (lysine methyltransferase 3C/SET and MYND domain-containing 2)-mediated monomethylation on K370 (P53K370me_1_) reduces the activity of P53 [25, 26]. Interestingly, KMT7/SETD7-mediated P53K372me_1_ reduces the interaction between P53 and KMT3C/SMYD2 and as a consequence the KMT3C/SMYD2-mediated P53K370me_1_ finally increasing the transcriptional activity of P53 [25]. Altogether, these observations suggest a competing mechanism between different KMTs to differentially regulate the activity of P53. Of note, KMT7/SETD7-mediated P53K372me1 is a prerequisite for KAT3B/EP300-mediated acetylation of P53 on K373 and K382 [27] which play a positive role in the P53-mediated transcriptional response [28–31]. In line with this, KMT7/SETD7-depleted mouse cells fail to methylate P53 on K369 (corresponding to human 372) and fail to acetylate P53 on K379 [corresponding to human 382 [32]]. Mechanistically, P53K369me_1_ acts as a docking site for the recruitment of the histone acetyltransferase KAT5/TIP60 (lysine acetyltransferase 5/tat interacting protein 60 kDa) [32] that is known to positively impact on the P53-dependent apoptotic response following genotoxic stress [33, 34]. KMT7/SETD7 regulates P53-dependent transcription also via an indirect mechanism that involves sirtuin 1 (SIRT1). SIRT1 is known to deacetylate P53 reducing its activity [35] however, upon DNA damage, KMT7/SETD7 methylates SIRT1 releasing the SIRT1-P53 interaction finally leading to increased P53-dependent response [36]. The same K370 of P53 is dimethylated (P53K370me_2_) by an unknown methyltransferase [37] but in contrast to P53K370me_1_, P53K370me_2_ plays a positive role in the transcriptional activity of P53 and this occurs via the binding of the coactivator tumor protein P53 binding protein 1 (TP53BP1) to P53K370me_2_ [37] and the Tudor domain of PHD finger protein 20 (PHF20) [38]. Of note, P53K370me_2_ is demethylated by KDM1A/LSD1 [37].

Additional lysine residues within P53 are known to be methylated: For example, KMT5A/SETD8 (lysine methyltransferase 5A/SET domain-containing 8) monomethylates P53 on K382 (P53K382me_1_) reducing its DNA binding and transcriptional activity and KMT5A/SETD8 depletion leads to increased transcriptional activity of P53 in response to DNA damages [26, 39]. Mechanistically, P53K382me_1_ acts as a docking site for the MBT domains of lethal(3)malignant brain tumor-like protein 1 (L3MBTL1) which depletion leads to increased P53-dependent transcriptional response upon DNA damage [40]. Interestingly, reduction in P53K382me_1_ is associated with increased acetylation on K382 which supports P53 transcriptional activity [39]. The same residue has been described to be dimethylated (P53K382me_2_) by unknown KMT(s) and, under these circumstances, to be bound by the methyl reader and coactivator TP53BP1 [41]. P53K382me_2_ increases upon DNA damages [41] and the positive effect of TP53BP1 on P53 via K382me_2_ is downmodulated by Tudor-interacting repair regulator (TIRR) which inhibits the interaction between methylated P53 and the Tudor domain of TP53BP1 [42]. P53K382me_2_ is also bound by the Tudor domain of PHF20 leading to reduced MDM2-mediated P53 ubiquitination finally reducing its degradation and as a consequence increasing stability and activity of P53 [38]. Furthermore, K373 is dimethylated (and probably also monomethylated) by KMT1C/EHMT2/G9A (lysine methyltransferase 1C/euchromatic histone-lysine *N*-methyltransferase 2) and KMT1D/EHMT1/GLP (lysine methyltransferase 1D/euchromatic histone-lysine N-methyltransferase 1/ G9a-like protein 1) and their knockdown results in increased apoptosis suggesting that the KMT1C/EHMT2/G9A- and KMT1D/EHMT1/GLP-mediated methylation of P53 reduces its transcriptional activity [43]. Finally, structural studies suggest KMT2 (lysine methyltransferase 2) family members as possible K methyl writers within P53 [44] however, more efforts are required to support this notion.

**Fig. 3:**
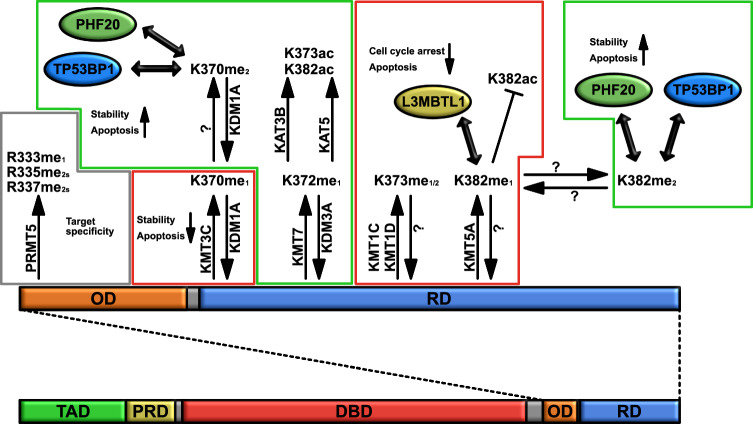
Regulation of tumor suppressor P53 by lysine (K) and arginine (R) methylation. Lysine (K) methylation of the tumor suppressor P53 occurs at several lysineresidues located within the regulatory domain (RD). KMT3C/SMYD2 (lysine methyltransferase 3C/ SET and MYND domain-containing 2) monomethylates K370 (P53K370me_1_) leading to reduced activity of P53 and apoptosis. An unknown lysine methyltransferase (KMT) further dimethylates K370 (P53K370me_2_) which is recognized by Tudor domain-containing proteins [tumor protein P53 binding protein 1 (TP53BP1) or PHD finger protein 20 (PHF20), leading to increased P53 stability and apoptosis. KMT7/SETD7 monomethylates K372 (P53K372me_1_) and this supports both the KAT3B/EP300 (lysine acetyltransferase 3B/E1A-binding protein 300 kD)-mediated acetylation on K373 and K382 and the KAT5/TIP60 (lysine acetyltransferase 5/tat interacting protein 60 kDa)-mediated acetylation of P53. Furthermore, KMT7/SETD7-mediated P53K372me_1_ reduces the interaction between P53 and KMT3C/SMYD2 and as consequence it reduces the KMT3C/SMYD2-mediated P53K370me_1_. As a consequence, P53K372me_1_ increases stability of P53 and apoptosis. Both P53K370me_1_ and P53K370me_2_ are demethylated by KDM1A/LSD1 (lysine demethylase 1A/lysine-specific histone demethylase 1). KMT1C/EHMT2/G9A (lysine methyltransferase 1C/euchromatic histone-lysine N-methyltransferase 2) and KMT1D/EHMT1/GLP (lysine methyltransferase 1D/euchromatic histone-lysine N-methyltransferase 1/G9a-like protein 1) methylate K373 on P53 leading to reduced P53 transcriptional activity and apoptosis. KMT5A/SETD8 (lysine methyltransferase 5A/SET domain-containing 8) monomethylates P53 on K382 (P53K382me_1_) reducing acetylation on K382 and acting as a docking site for the methyl binding domains (MBT) of lethal(3)malignant brain tumor-like protein 1 (L3MBTL1) finally leading to reduced P53 transcriptional activity, cell cycle arrest and apoptosis. P53K382me_1_ is further dimethylated (P53K382me_2_) by unknown KMTs and this post-translation modification (PTM) is further bound by Tudor domain-containing proteins (TP53BP1 or PHF20) increasing stability of P53 and apoptosis. Arginine (R) methylation of P53 occurs on R333 (monomethylation, R333me_1_), R335 (symmetric dimethylation, R335me_2s_) and R337 (symmetric dimethylation, R337me_2s_) and these methylations regulate the target specificity of P53. Green and red boxes indicate methylation events that play a positive or negative role in the P53-dependent response, respectively. Gray box indicates methylation events that regulate the target specificity of P53 response. *DBD* DNA binding domain; *KAT3B = KAT3B/EP300* lysine acetyltransferase 3B/E1A-binding protein 300 kD; *KAT5 = KAT5/TIP60* lysine acetyltransferase 5/tat interacting protein 60 kDa; *KDM1A = KDM1A/LSD1* lysine demethylase 1A/lysine-specific histone demethylase 1; *KDM3A = KDM3A/JMJD1A* lysine demethylase 3A/Jumonji domain-containing 1A; *KMT1C = KMT1C/EHMT2/G9A* lysine methyltransferase 1C/euchromatic histone-lysine N-methyltransferase 2; *KMT1D = KMT1D/EHMT1/GLP* lysine methyltransferase 1D/euchromatic histone-lysine *N*-methyltransferase 1/ G9a-like protein 1; *KMT3C = KMT3C/SMYD2* lysine methyltransferase 3C/SET and MYND domain-containing 2; *KMT5A = KMT5A/SETD8* lysine methyltransferase 5A/SET domain-containing 8; *KMT7 = KMT7/SETD7* lysine methyltransferase 7/SET domain-containing 7; *L3MBTL1* lethal(3)malignant brain tumor-like protein 1; *OD* oligomerization domain; *PHF20* PHD finger protein 20; *PRD* proline-rich domain; *PRMT5* protein arginine methyltransferase 5; *RD* regulatory domain; *TAD* transcriptional activation domain; *TP53BP1* tumor protein P53 binding protein 1

Mass-spectrometry studies identified P53 methylation also on arginine residues: R335 and R337 are symmetrically dimethylated (P53R335me_2s_ and P53R337me_2s_), whereas R333 is monomethylated [P53R333me_1_ [45]]. Arginine methylation of P53 is catalyzed by PRMT5 and this methylation impact on the transcriptional activity of P53 even if with variabilities among its different transcriptional targets [45]. In summary, lysine and arginine methylation of P53 has a profound and different impact on the stability, and consequently activity, of P53 based on the specific residues that are modified, on the degree of methylation as well as on the methyl reader protein involved (Fig. 3).

### E2F1

Another key tumor suppressor is retinoblastoma protein (pRB) which binds E2F family of transcription factors preventing their activity [46]. E2F family members promote cell cycle as well as apoptosis [47] and are regulated by several different PTMs. Among them, monomethylation on K185 of E2F1 (E2F1K185me_1_) is dynamically regulated by KMT7/SETD7 and KDM1A/LSD1 [48, 49] and reduces the stability of E2F1 protein via an ubiquitin-dependent mechanism finally downregulating the proapoptotic activity of E2F1 [48, 50]. Furthermore, E2F1K185me_1_ crosstalks with other PTMs reducing both acetylation and phosphorylation of E2F1 itself upon DNA damages [48, 50]. In contrast to this, Xie and colleagues observed that KMT7/SETD7-mediated K185me_1_ of E2F1 stabilizes the protein itself and increases cell death upon DNA damages [49]. The reasons for these discrepancies are not clear but they might depend on the cell type and/or DNA damage agent used in the two different studies [48, 49]. Interestingly, SETD6 (SET domain-containing 6) expression has been shown to be regulated by E2F1 and SETD6 monomethylates E2F1 on K117 [E2F1K117me_1_ [51]] increasing the DNA binding of E2F1 at the *SETD6* locus finally supporting its expression in a way to establish a positive feedback loop [51].

On the other side, symmetric arginine methylation of E2F1 is promoted by PRMT5 which predominantly methylates R111 and R113 [E2F1R111me_2s_ and E2F1R113me_2s_ [52, 53]]. Symmetric arginine methylated E2F1 is bound by the Tudor domain of p100-Tudor staphylococcal nuclease (TSN) reducing E2F1 stability and apoptosis [53]. In line with this, a methylation-defective arginine E2F1 mutant is more stable, less ubiquitinated, has a higher transcriptional activity and reduces cell growth compared to its wildtype counterpart and similar results are also observed upon knockdown of PRMT5 [52, 53]. Interestingly, reduced symmetric arginine methylation of E2F1 is detected upon DNA damages and this leads to stabilization of E2F1 and increased apoptosis [52]. Asymmetric arginine methylation of E2F1 has been also described, it occurs on R109 (E2F1R109me_2a_) and is catalyzed by PRMT1 [53]. A methylation-defective R109 mutant of E2F1 or PRMT1 knockdown result in reduced stability of E2F1, reduced transcription of E2F1 target genes and increased cell growth [53]. Interestingly, PRMT1 knockdown leads to reduced apoptosis upon DNA damage [53] while PRMT5 knockdown has the opposite effect [52, 53]. This is explained by the observation that PRMT1-mediated E2F1 methylation prevents the PRMT5-mediated one and binding of CYCLIN A to E2F1 blocks the PRMT1-mediated methylation stimulating the PRMT5-mediated one, keeping cells in cell cycle [53] proposing a competing mechanism for the methylation of E2F1 by the two PRMTs. In summary, these studies reveal that PRMT1-dependent asymmetric arginine methylation of E2F1 prevents its PRMT5-dependent symmetric arginine methylation promoting apoptosis upon DNA damages. CYCLIN A binding to E2F1 prevents asymmetric arginine methylation augmenting symmetric arginine methylation and recruiting p100-TSN which finally leads to cell survival and proliferation.

### Nuclear receptors (NRs)

Nuclear receptors (NRs) are the key mediators of hormone signaling. Generally, ligand binding to NRs [for example estrogen receptor alpha (ERα)] in the cytoplasm leads to their nuclear translocation finally activating expression of target genes. Alternatively, some NRs dimerize already in absence of ligand acting as repressor of transcription and ligand binding converts them in transcriptional activators. This is the case for example of retinoic acid receptor alpha (RARα) heterodimerizing with retinoid X receptor (RXR).

ERα is monomethylated on K302 (ERαK302me_1_) by KMT7/SETD7 [54, 55], having a positive impact on the stability of ERα, on its ligand-dependent transcriptional activity and on its DNA binding [54]. Interestingly, acetylation of the same residue has been proposed to attenuate the ERα transcriptional response [56], suggesting that different PTMs of the same residue within ERα are used in a competitive fashion to modulate its activity. On the other hand, KMT3C/SMYD2-dependent monomethylation of ERα on K266 (ERαK266me_1_) has a negative impact on gene transcription [57] and is enhanced by the chaperones heat shock protein 90 (HSP90)-P23 [58]. KMT3C/SMYD2 depletion leads to increased DNA binding of ERα associated with increased gene transcription of its target genes upon estradiol (E_2_) treatment. However, this depletion has no impact on H3K4me_2_ while it leads to increased H3K4me_3_ signal [57]. Of note, ERαK266me_1_ is demethylated by KDM1A/LSD1 and importantly, it prevents the KAT3B/EP300 and KAT3A/CBP (lysine acetyltransferase 3A/CREB-binding protein)-mediated acetylation of ERα at K266 and K268 [57] which play a positive function in the activity of the receptor [59]. ERα is also asymmetrically dimethylated within its DNA binding domain on R260 (R260me_2a_) by PRMT1 [60]: ERαR260me_2a_ occurs in the cytoplasm, is required for its E_2_-induced interaction with SRC and p85, a subunit of the phosphoinositide 3-kinase (PI3K) [60] and this arginine methylation has been suggested to be removed by JMJD6 [9] even if the JMJD6 arginine demethylase activity is still under debate.

Trimethylation at K347 within RARα (RARαK347me_3_) has a positive impact on its ligand-dependent transcriptional activity facilitating its interaction with both its heterodimeric partner RXR and the transcriptional coactivator KAT2B/PCAF (lysine acetyltransferase 2B/EP300-CBP-associated factor) [61]. Interestingly, in absence of ligand, RARαK347me_3_ supports its interaction with the corepressor nuclear receptor corepressor 1 (NCoR1) [61]. Two additional monomethylated lysine residues have been identified within RARα: K109 (RARαK109me_1_) within the DNA-binding domain (DBD) and K171 (RARαK171me_1_) within the hinge region [62]. Mutational analysis revealed that RARαK109me_1_ but not RARαK171me_1_ is required for ligand-dependent transcriptional activation and surprisingly RARαK109me_1_ has a stronger impact, compared to RARαK347me_3_, on the transcriptional activity of RARα [62]. Similarly to RARαK347me_3_, RARαK109me_1_ is required for the interaction of RARα with RXR and KAT2B/PCAF [62].

Retinoic acid receptor–related orphan receptor alpha (RORα) is also regulated by methylation of both lysine and arginine residues. KMT6A/EZH2 (lysine methyltransferase 6A/enhancer of zeste homolog 2) monomethylates RORα at K38 (RORαK38me_1_) creating a docking site that is recognized by the adaptor protein DCAF1 [damage-specific DNA binding protein 1 (DDB1)-cullin4 (CUL4)-associated factor] which bridges RORα to the DDB1/CUL4 E3 ubiquitin ligase complex promoting ubiquitination and proteasomal-dependent degradation of RORα [63]. As a consequence, RORαK38me_1_ is unstable and this is reflected on its DNA binding and transcriptional activity [63]. Further modifications of RORα include PRMT5-mediated methylation on R37 which acts as a docking site for Itchy E3 ubiquitin protein ligase (ITCH) promoting the ubiquitin-dependent proteasomal degradation of RORα [64].

Lysine monomethylation of progesterone receptor (PR) occurs within the activation function 1 (AF-1) domain at K464 (PRK464me_1_) and this modification potentially reduces the transactivation activity of the receptor [65]. On the other side, PR is also asymmetrically dimethylated by PRMT1 on R637 (PRR637me_2a_) upon progesterone treatment and PRMT1 expression reduces the stability of PR [66], suggesting that PRMT1-mediated methylation of PR plays a negative role in regulating its stability.

Similarly to PR methylation on K464, KMT7/SETD7-dependent methylation of androgen receptor (AR) at K630 is required for efficient gene induction upon stimulation with the synthetic androgen R1881 positively influencing both its DNA binding and the recruitment of KAT2B/PCAF [67]. A second study could support the notion that KMT7/SETD7-dependent methylation of AR plays a positive role in its transcriptional activity. However, in this case, the authors could detect methylation on K632 but not on K630 [68]; In contrast, Ko and colleagues could detect methylation on K630 but not on K632 [67]. The reason for this discrepancy remains however unclear. KMT3C/SMYD2 interacts with AR and methylates it at unknown residue(s) [69]. KMT3C/SMYD2 increases the stability of AR reducing its ubiquitination and knockdown of KMT3C/SMYD2 reduces its DNA binding and the expression of target genes [69]. Finally, PRMT5-dependent arginine methylation (both ωMMAs and ωsDMA) of AR has also been described and it occurs on R761 leading to reduced DNA binding and expression of its target genes [70].

### NF-κB

One of the key mediators of the inflammatory response is the transcription factor nuclear factor 'kappa-light-chain-enhancer' of activated B-cells (NF-κB). NF-κB consists of either homo- or heterodimers of different subunits that include P50, P52, RELA, RELB and C-REL [71]. KMT7/SETD7 monomethylates RELA at K37 (RELAK37me_1_) and this methylation is required for both DNA binding and induction of a subset of RELA target genes [*tumor necrosis factor alpha* (*TNFα*) and *C-X-C motif chemokine 10* (*CXCL10*)] in response to TNFα or interleukin 1β (IL1β) stimulation [72, 73]. In addition, KMT7/SETD7-dependent monomethylation of RELA at K314 and K315 (RELAK314me_1_ and RELA K315me_1_) reduces gene induction upon TNFα stimulation by increasing RELA ubiquitination and as consequence its stability [74]. Interestingly, KAT3B/EP300-mediated acetylation on K310 of RELA reduces its interaction with KMT7/SETD7 and, as a consequence, monomethylation of RELA on K314 and K315 and finally its ubiquitination [75]. K310 of RELA is also monomethylated (RELAK310me_1_) by SETD6 and this methylation reduces the transcriptional activity of RELA upon stimulation with TNFα and RELA-dependent cell proliferation [76]. Mechanistically, RELAK310me_1_ acts as a docking site for the recruitment of KMT1D/EHMT1/GLP which promotes H3K9me_2_ and this interaction is prevented by protein kinase C zeta (PKCζ)-mediated phosphorylation on serine 311 of RELA [76, 77].

Additional methylated lysine residues have been identified within RELA. For example, monomethylation at K218 (RELAK218me_1_) and dimethylation at K221 (RELAK221me_2_) are dynamically regulated by KMT3B/NSD1 (lysine methyltransferase 3B/nuclear receptor–binding SET domain-containing protein 1) and KDM2A/JHDM1A (lysine demethylase 2A/JmjC domain-containing histone demethylation protein 1A) in response to TNFα or IL1β stimulation and this methylation has a positive impact on the expression of a subset of RELA target genes and stimulates cell proliferation [73, 78]. Interestingly, IL1β stimulation leads to increased expression of KDM2A/JHDM1A [78] suggesting a possible negative feedback loop that regulates the RELA-dependent response.

PRMT5 symmetrically dimethylates RELA at R30 (RELAR30me_2s_) upon stimulation with IL1β increasing its transcriptional activity [79]. A crystal-structure-based model suggests that RELAR30me_2s_ may increase its DNA binding [79]. R30 of RELA is also asymmetrically dimethylated by PRMT1 reducing its DNA binding activity in response to stimulation with TNFα [80]. Those studies suggest that different arginine methylation, symmetric vs asymmetric, have a different impact on the RELA-dependent transcriptional response. Another study could show that more arginine residues within RELA are methylated and that those different residues have a different impact on the transcriptional activity of RELA in a gene-specific fashion [81, 82]. Finally, PRMT4-mediated monomethylation of RELA is required for neuronal differentiation from embryonic stem cells (ESCs) however, the exact monomethylated residue(s) have not yet been identified [83].

### STAT3

Cytokines like IL6 bind to membrane receptors triggering the activation of Janus kinases (JAKs), which phosphorylate signal transducer and activator of transcription 3 (STAT3) on Y705 (STAT3Y705p). This phosphorylation promotes the homodimerization of STAT3, leading to its release from the receptor, followed by nuclear translocation and activation of target genes [84]. STAT3 is dimethylated on K140 (STAT3K140me_2_) by KMT7/SETD7 and demethylated by KDM1A/LSD1 [85]. Interestingly, STAT3K140me_2_ occurs in the nucleus, requires DNA binding, significantly increases upon stimulation with interleukin 6 (IL6) and is required for the induction of only a subset of target genes [85]. In addition to KDM1A/LSD1, also KDM3C/JMJD1C (lysine demethylase 3C/Jumonji domain-containing 1C) is able to demethylate STAT3 on K140 [86, 87]. Mechanistically, KDM3C/JMJD1C-mediated STAT3 demethylation supports the interaction of STAT3 with protein tyrosine phosphatase non-receptor type 6 (PTPN6) and, as a consequence, it reduces STAT3 phosphorylation and activity [86]. Additionally, in colon tumor cells, STAT3 is dimethylated on K49 (STAT3K49me_2_) by KMT6A/EZH2 and this methylation is required for gene expression, increases upon IL6 stimulation and it is dependent on phosphorylation on Y705 [88]. In glioblastoma cells, KMT6A/EZH2 trimethylates STAT3 on K180 (STAT3K180me_3_) playing again a positive role in the STAT3-dependent transcriptional response and supporting STAT3Y705p [89].

The activity of STAT3 is also regulated by arginine methylation: PRMT6 asymmetrically dimethylates R729 of STAT3 (STAT3R729me_2a_) and this mark positively correlates with STAT3Y705p [90]. Mechanistically STAT3R729me_2a_ positively influences its interaction with JAK2 and its membrane localization [90]. Importantly, STAT3R729me_2a_ positively correlates with PRMT6 protein levels and STAT3Y705p levels in breast cancer tissues and patients with high levels of STAT3R729me_2a_ have a lower overall survival [90]. Notably, STAT3R729me_2a_ plays an important role also in PRMT6-mediated tumor metastasis and PRMT6 inhibition is able to reduce metastasis [90]. In addition to this, STAT3 is methylated by PRMT2 on R31 and PRMT2 depletion leads to increased STAT3Y705P upon leptin treatment which is known to induce STAT3 signaling [91] suggesting that methylation of R31 within STAT3 plays a negative role on its phosphorylation on Y705. Interestingly, PRMT2 knockout mice are lean, less prone to develop diet-induced obesity and have reduced glycogen stores suggesting that PRMT2-dependent methylation of STAT3 on R31 plays a positive role in obesity [91]. Finally, TNFα-induced protein 8-like protein 1 (TIPE1) inhibits osteosarcoma carcinogenesis and metastatic activity by binding to and inhibiting PRMT1 which is required for the asymmetric dimethylation of STAT3 on R688 (STAT3R688me_2a_), a modification that stimulates the activity of STAT3 itself [92].

### Additional examples

Other examples of transcription factors methylated on lysine and/or arginine residues include Yin Yang 1 (YY1), octamer-binding protein 4 (OCT4), runt-related transcription factor 1 (RUNX1) and 3 (RUNX3), myocyte enhancer factor 2D (MEF2D) and GLI proteins.

Yin Yang 1 (YY1) is a zinc-finger transcription factor that acts as both a repressor and an activator of transcription. KMT7/SETD7 monomethylates YY1 on K173 and K411 (YY1K173me_1_ and YY1K411me_1_, respectively) promoting its DNA binding and influencing its transcriptional activity [93]. Several additional lysine residues within YY1 have been described to be methylated and importantly, di- and trimethylation on K258 are demethylated by KDM7B/PHF8 (lysine methyltransferase 7B/PHD finger protein 8) [94]. KDM7B/PHF8 depletion as well as K258R mutation within YY1 lead to its reduced DNA binding associated with changes in gene expression [94]]. Similarly to YY1, also its homolog YY2 has been described to be monomethylated on K247 [YY1K247me_1_ [95]]. This methylation is regulated by KMT7/SETD7 and KDM1A/LSD1 and again it influences DNA binding and transcriptional activity of YY2 [95].

RUNX proteins are members of a family of transcription factors involved in several developmental decisions. The DNA binding RUNX factors heterodimerize with the non-DNA binding protein core-binding factor beta (CBFβ) which function is to stabilize the RUNX/DNA interaction [96]. While RUNX1 is required for hematopoiesis, RUNX2 is needed for osteogenesis and RUNX3 for neurogenesis and other developmental processes [96]. Under hypoxia, RUNX3 is methylated (di- and/or monomethylated) on K129 and K171 by KMT1C/EHMT2/G9A leading to reduced transactivation activity of RUNX3 via inhibition of its interaction with KAT3B/EP300 and CBFβ and reducing, as a consequence, acetylation of RUNX3 itself which is required for its nuclear import [97]. Another member of this family of transcription factor, RUNX1 [also known as acute myeloid leukemia 1 (AML1)], has been described to interact with and to be methylated on R206 and R210 by PRMT1 [98]. This arginine methylation within RUNX1 prevents its interaction with the transcriptional corepressor SIN3A and methylation-defective arginine mutants of RUNX1 have an increased transcriptional activity compared to their wild type counterpart [98, 99]. Making use of genetic mouse models, it was possible to determine that arginine methylation within RUNX1 has no role for definitive hematopoiesis and steady-state thrombopoiesis but it impacts of the peripheral CD4^+^ T-cells [99] and increases resistance to apoptosis of hematopoietic stem cells (HSCs) upon genotoxic stress [100]. RUNX1 is also frequently mutated in leukemia and for example, it is fused to the corepressor eight-twenty-one (ETO) generating the oncofusion protein AML1/ETO in acute myeloid leukemia (AML). PRMT1-mediated arginine methylation of AML1/ETO has also been described within the “AML1” portion of the protein and PRMT1 depletion leads to reduced transcription of AML1/ETO targets and compromises the self-renewal ability of AML1/ETO [101].

Mass spectrometry (MS) has identified several methylated lysine residues within the transcription factor OCT4 which is a master transcription factor for ESCs [102]. Monomethylation on K222 of OCT4 (OCT4K222me_1_) is regulated by KDM1A/LSD1 which promotes proteasome-independent degradation of OCT4 [102]. Another master transcription factor for stemness, sex-determining region Y protein (SRY)-box transcription factor 2 (SOX2), is methylated on lysine residues. KMT7/SETD7-mediated monomethylation of mouse SOX2 on K119 (SOX2K119me_1_; corresponding to human 117) promotes ubiquitination and proteasomal degradation of SOX2 and reduces its transcriptional activity and interaction with KAT3B/EP300 [103]. Mechanistically, SOX2K119me_1_ acts as a docking site for the recruitment of the E3 ubiquitin ligase WW domain-containing E3 ubiquitin protein ligase 2 (WWP2) via its E6-AP carboxyl terminus (HECT) domain and interestingly, this methylation counteracts AKT serine/threonine kinase 1 (AKT1)-mediated phosphorylation on T118 which, in contrast to SOX2K119me_1_, promotes SOX2 stabilization [103]. KMT7/SETD7 also monomethylates human SOX2 on K42 (SOX2K42me_1_) and both methylation sites (human K42 and K117) are demethylated by KDM1A/LSD1 promoting SOX2 stability [104]. In addition, both methylation sites (human K42 and K117) function as docking sites for PHD finger protein 20 like 1 (PHF20L1) which finally protects SOX2 from proteolysis [104]. SOX2 is also methylated on R113 by PRMT4 promoting SOX2 self-association [105].

MEF2D is another good example of lysine methylation of non-histone proteins. MEF2D plays important roles in hematopoiesis as well as muscle and neuronal development [106]. KMT1C/EHMT2/G9A dynamically monomethylates MEF2D on K267 (MEF2DK267me_1_) and this methylation is counteracted by KDM1A/LSD1 [107]. Interestingly, MEF2DK267me_1_ is reduced during differentiation of C2C12 cells and this reduced methylation is associated with increased DNA binding and transcriptional activity of MEF2D [107].

One further example is represented by GLI3, one of the key transcriptional mediator of the Hedgehog (HH) signaling pathway. GLI3 is proteolytically cleaved in absence of HH signals acting as a transcriptional repressor however, in presence of HH signaling, it is stabilized and functions as a transcriptional activator [108]. The full-length but not the proteolytically processed GLI3 protein is monomethylated on K436 and K595 (GLI3K436me_1_ and GLI3K595me_1_) by KMT7/SETD7 [109]]. This methylation promotes the HH-mediated gene expression via two different mechanisms: Methylation on K436 increases GLI3 protein stability while methylation on K595 promotes its DNA binding [109]. Interestingly, methylation of GLI3 has a positive effect on both proliferation and migration of lung cancer cells [109]. Another member of the GLI family, GLI1, is asymmetrically dimethylated on R597 by PRMT1 and this methylation plays a positive role in the GLI1 transcriptional activity by promoting its DNA binding ability finally increasing its oncogenic activity [110]. Finally, GLI1 is also symmetrically dimethylated on R515, R990 and R1018 by PRMT5 and these methylation events (with exclusion of R515) stabilize the GLI1 protein inhibiting its ITCH/NUMB-mediated ubiquitination [111].

## Methylation of DNA methyltransferases (DNMTs)

Methylation of the DNA base cytosine is a key determinant in regulating gene expression and is mediated by DNA methyltransferases (DNMTs). There are five human DNMTs isoforms: DNMT1, DNMT3A and DNMT3B but not DNMT2 and DNMT3L have DNMT catalytic activity. DNMT1 is required for maintenance of DNA methylation and is responsible of post-replicative copy of DNA methylation patterns from parental strands into newly synthesized DNA. On the other side, DNMT3A and DNMT3B are the so-called de novo DNMTs which are responsible to establish new methylation patterns. The epigenetic information represented by methylated DNA is subsequently read via the methyl-DNA binding domain (MBD) which bridges methylated DNA to corepressors such as histone deacetylases (HDACs).

DNMT1 is monomethylated on K142 (DNMT1K142me_1_) by KMT7/SETD7 and this methylation promotes the proteasome-dependent degradation of DNMT1 [50, 112]. Interestingly, KMT7-SETD7-mediated DNMT1K142me_1_ is prevented by AKT1-mediated phosphorylation at S143, a mark that increases the stability of DNMT1 [113]. Additionally, KMT7/SETD7 and KDM1A/LSD1 dynamically regulate the methylation state of DNMT1 on K1096 and KDM1A/LSD1 depletion in ESCs leads to growth and differentiation defects associated with DNA hypomethylation and reduced DNMT1 protein levels [114].

KMT1C/EHMT2/G9A and KMT1D/EHMT1/GLP dimethylate human DNMT3A on K47 creating a docking site that is recognized by the chromodomain of M-phase phosphoprotein 8 (MPP8) [115]. Given that KMT1D/EHMT1/GLP auto-methylates at K205 and that this methylated site is again bound by the chromodomain of MPP8 and given that MPP8 can dimerize, this suggest a simple model to explain how H3K9 methylation and DNA methylation are established in combination together on chromatin [115].

The methyl-DNA binding reader MBD2 is methylated on R residues by PRMT1 and PRMT5 reducing its interaction both with HDACs and methylated DNA [116], suggesting that not only methylation of DNMTs but also of methyl DNA readers can contribute to heterochromatin regulation.

## Translational aspects

Since the regulation of transcription factors is also dysregulated in pathological conditions like cancer, the search for inhibitors of methyltransferase or demethylase is a logical next step. We briefly mention a few examples and the reader is referred to other reviews focusing on this topic [117].

Tazemetostat is an inhibitor of KMT6A/EZH2 and recently entered clinical trials. Tazemetostat is an orally bioavailable potent and selective SAM competitive KMT6A/EZH2 inhibitor. In preclinical models Tazemetostat is potentially effective in the context of non-Hodgkin lymphoma [118], malignant rhabdoid tumors [119] and synovial sarcoma [120]. Based on these results, Tazemetostat has been used in a phase-II study for the treatment of malignant pleural mesothelioma patients characterized by BAP1 [breast cancer 1 (BRCA1) associated protein 1] inactivation [121] showing a good tolerability to the drug. The promising efficacy of Tazemetostat has been further marked in relapsed/refractory follicular lymphomas and diffuse large B-cell lymphomas [122–125].

Inhibition of KMT4/DOT1L (lysine methyltransferase 4/disrupter of telomeric silencing 1-like) deserved also attention in more clinical trials. Pinometostat has been used in a phase 1 study mainly focused on acute leukemia patients characterized by mixed lineage leukemia (*MLL*) gene rearrangements [(MLL-r), 11q23 translocations; [126]]. This study could demonstrate that even if the drug was well tolerated, only 2 out of the 51 patients enrolled in the study showed a complete remission [126]. A second phase I study conducted on MLL-r children supported again the safety of the drug however no drug response was observed [127].

Furthermore, KDM1A/LSD1 inhibitors have been tested in clinical trials, for example Iadademstat has been used in combination with azacytidine for the treatment of AML patients showing a manageable safety with promising efficacy [128]. In addition, the KDM1A/LSD1 inhibitor TAK-418 has been tested for tolerability, pharmacokinetics and pharmacodynamics in Kabuki syndrome patients with promising results [129].

## Perspectives

Methylation, compared to other PTMs such as phosphorylation and ubiquitination, tends to be relatively slow and stable. A key aspect in regard to mechanism for methylation of lysine residues is that methylation prevents ubiquitination of the same lysine residue and as consequence protein turnover. Alternatively, it is also possible that methylation on a lysine residue supports ubiquitination of a different lysine residue supporting protein turnover. It will be interesting to investigate whether this also affects local concentration of proteins as for example often suggested for nuclear condensates with specialized functions such as transcriptional repression or microscopically visible nuclear speckles. Arginine methylation is frequently identified in proteomic studies as affecting RNA-binding proteins involved in post-transcriptional regulation. It will be interesting to explore whether arginine methylated transcription factors play a role not only in transcriptional regulation but also in processes like splicing and/or nuclear export.

By now we know that methylation of transcription factors can regulate their stability/turnover as well the binding to their interaction partners. Those partners can be enzymes that regulate both chromatin structure as well as PTMs within the transcription factors themselves. Alternatively, interaction partners can be readers of PTMs or scaffold and architectural proteins. In this case, PTMs of transcription factors can modulate gene transcription by a several different mechanisms regulating even chromatin looping giving finally gene specificity. In addition, environmental factors such as oxygen levels profoundly affect methylation states, as observed for HIF transcription factors. This in turn affects development and differentiation.

Aberrant transcription factor methylation can be caused by mutations and/or aberrant activity of writers, readers and erasers leading to diseases such as cancer but also genetic diseases. In future, the development of methyl-specific antibodies is are needed as tools to properly investigate and understand the link between aberrant methylation of transcription factors and pathological conditions. Such methylation-specific antibodies could also be useful reagents for clinical diagnostics. In parallel, the development of specific small molecule inhibitors especially of writers and erasers could be also extremely valuable not only to understand the molecular mechanism but also for clinical applications.

In addition, it remains to be seen how extremely high oxygen levels, that is hyperoxia, affect methylation states of transcription factors. Hyperoxia could affect the expression levels of writers, erasers and readers, but also could affect enzymatic activities especially of the demethylases as for example the activity of the O_2_-dependent JMJC domain-containing family of KDMs. Based on this, it has been shown that hyperoxia influences the expression of KMT6A/EZH2 [130] however, it is unknown whether this hyperoxia-induced KMT6A/EZH2 downregulation impacts on the methylation of HIF proteins.

Similarly to proteomic approaches investigating kinases or E3 ubiquitin ligases, it would be attractive to perform loss-of-function of the different enzymes involved in controlling lysine and arginine methylation. This can be combined with subsequent pan-methyl-lysine or pan-methyl-arginine antibodies immunoaffinity purification and mass-spectrometry [131–133], in order to identify the specific sites that are regulated by each individual enzyme. This could significantly accelerate the discovery of new methylation substrates. High quality antibodies recognizing specific methylated residues of transcription factors are urgently needed to better study the role of lysine/arginine methylation of transcription factors for example making use of genomic approaches such as chromatin immunoprecipitation followed by deep sequencing (ChIP-Seq).

In addition, more efforts are definitely required to further characterize the arginine demethylase activity of RDM proteins and their activity in regard to transcription factors as substrates with obvious potential clinical implications.

The development of highly specific inhibitors versus KMTs, PRMTs and KDMs as well as methyl-binding readers is crucial not only to study the function of arginine/lysine methylation of transcription factors but also for their possible clinical use to treat diseases characterized by alteration of arginine/lysine methylation of transcription factors.

## Data Availability

Not applicable.
